# Protective Effects of Nutria Bile against Thioacetamide-Induced Liver Injury in Mice

**DOI:** 10.1155/2019/6059317

**Published:** 2019-06-25

**Authors:** Joo-Yeon Kong, Seong-Chan Yeon, Hu Jang Lee, Changkeun Kang, Jin-Kyu Park, Kyu-Shik Jeong, Il-Hwa Hong

**Affiliations:** ^1^Department of Pathology, College of Veterinary Medicine, Gyeongsang National University, Jinju, Republic of Korea; ^2^Department of Veterinary Clinical Sciences and Research Institute for Veterinary Science, College of Veterinary Medicine, Seoul National University, Seoul, Republic of Korea; ^3^Institute of Animal Medicine, Gyeongsang National University, Jinju, Republic of Korea; ^4^Department of Veterinary Pathology, College of Veterinary Medicine, Kyungpook National University, Daegu, Republic of Korea

## Abstract

Several eradication programs have been developed and executed to curb alien invasive species that tend to damage the ecological environments they colonize; however, only few studies have evaluated the utilization of carcasses of these species after eradication. Nutria (*Myocastor coypus*) is an invasive rodent species targeted by eradication programs in many countries. We noted that nutria produce large amounts of ursodeoxycholic acid (UDCA) in their bile. UDCA is a unique component responsible for the anti-inflammatory and hepatoprotective effects exerted by bear bile. Therefore, we sought to examine the medicinal utility of nutria carcasses by investigating the hepatoprotective effect of their bile in mice. C57BL/6 mice were injected with thioacetamide (TAA), which induced liver damage by increasing Kupffer cell infiltration. Administration of nutria bile reduced hepatic inflammation, improved hepatic function, and increased the levels of senescence marker protein 30 (an indicator of hepatocyte viability). Our results show that nutria bile exerts protective effects against TAA-induced liver injury in mice, suggesting that nutria carcasses may be used for the treatment of liver injuries.

## 1. Introduction

Alien invasive species are nonnative organisms that damage the ecological environment they colonize. Ecosystem disturbance due to invasive species is a serious problem as it contributes to high economic costs and loss of biological diversity. Economic losses caused by ecosystem disturbances account for billions of dollars each year in countries such as Australia, Brazil, Europe, India, New Zealand, South Africa, and the United States [1]. Global climate change and increase in international trade are expected to aggravate this problem [2]. To prevent the establishment of invasive species, many countries conduct eradication (complete removal) or control (long term reduction including containment) programs [3–5]. As carcass processing is costly, there is growing demand for the utilization, rather than disposal, of carcasses.

Nutria (*Myocastor coypus*) is a semiaquatic rodent that is native to South America [6]. In the 1980s, nutria was used for fur and meat production in Korea. Once the demand for these rodents declined, they were released into the wild and have since been labeled as pests because of their high rate of reproduction and the severe damage they cause to the farming industry [7, 8]. Currently, nutria is ranked in the top 100 alien invasive species [9]. According to the Korean Ministry of Environment (2014), an eradication program launched against invasive nutria amounted to an estimated 9.5 million dollars over a period of ten years [10].

Tint et al. reported that nutria bile contains approximately 37% ursodeoxycholic acid (UDCA) [11]. UDCA is considered a unique component of bear bile (belonging to the family Ursidae), present at a proportion of up to 39%. Interestingly, UDCA is not found in other carnivores and humans [12, 13]. In traditional Asian medicine, bear bile is used for its anti-inflammatory and hepatoprotective effects, which are attributed to the presence of UDCA [14, 15]. Since the hepatoprotective effect of synthetic UDCA has not been clearly established, the Federal Drug Administration (FDA) approved synthetic UDCA for the treatment of only one liver disease, primary biliary cirrhosis (PBC) [16]. To date, the therapeutic efficacy of synthetic UDCA for other liver diseases is controversial. Furthermore, approximately 40% of patients with PBC have shown suboptimal response to UDCA treatment [17]. However, only few experimental reports have been published regarding the hepatoprotective effect of total bile acid, with the exclusion of studies on the effects of synthetic UDCA; these latter investigations have been performed as bears are classified as endangered species in most countries [18]. The method of “milking” bile has also been controversial from an ethics standpoint.

Bile acids are synthesized from cholesterol in the liver and are considered to facilitate the absorption of dietary fats and lipid-soluble vitamins from the intestine. Recent studies have shown that bile acids regulate bile acid homeostasis, cholesterol metabolism, and the activation of nuclear receptors [19]. Bile acids contain conjugated forms of different types of cholic acids (CA) such as chenodeoxycholic acid (CDCA), deoxycholic acid (DCA), and lithocholic acid (LCA), with their composition varying among species [20]. Bear bile is composed of not only UDCA but also CDCA, CA, bile pigments, amino acids, fat, phospholipids, and various minerals [18, 20, 21]. It is unknown whether UDCA alone is hepatoprotective, or whether the therapeutic benefits of bear bile are attributable to the interaction between its various components. Therefore, we sought to determine the utility of nutria carcasses as an alternative source of bear bile. To elucidate this crucial information, we investigated the effects of nutria bile against TAA-induced liver injury in mice.

## 2. Materials and Methods

### 2.1. Bile Preparation

Wild nutria are captured and euthanized with CO_2_ gas and then the carcasses are incinerated as medical waste in accordance with a policy implemented by the Korean Ministry of Environment (KME). Among these, four were obtained for this study with the approval from the KME. Bile was withdrawn from their gallbladders with syringes, then pooled and lyophilized. The lyophilized bile powder was analyzed using high-performance liquid chromatography (HPLC) and was found to contain approximately 35% UDCA, 44.2% CDCA, and 21% 7-keto-LCA (Table 1). The pooled bile powder was diluted with 0.9% normal saline before administration to mice.

### 2.2. Experimental Design

Eight-week-old male C57BL/6 mice were purchased from Samtako (Osan, ROK) and fed standard chow and water* ad libitum*. Animals were housed at 22 ± 2°C, 50 ± 10% humidity, and under a 12-h light-dark cycle. All procedures were approved by the Animal Ethics Committee of Gyeongsang National University (Approval Number: GNU-151119-M0066) and conducted in compliance with the GNU guidelines for the care and use of laboratory animals. After a week of acclimation, mice were divided into six groups, each containing three to five mice: a control group (CON), a negative control group (NC), and four bile treatment groups (B10, B20, B50, and B100; Table S1). To induce liver injury, 50 mg/kg TAA was intraperitoneally injected every other day into mice in all groups except CON; this was performed thrice for six days. Bile solutions were administered at doses of 10 to 100 mg/kg once daily (five times in total) to mice in the four bile treatment groups. Mice were sacrificed 24 h after the last treatment by an overdose of isoflurane. Blood and liver tissues were then collected and stored at −80°C for further analysis.

### 2.3. Serum Chemistry

Blood samples were collected from caudal vena cava of mice under isoflurane anesthesia and centrifuged at 3,000 ×*g* for 15 min to obtain serum. Serum samples were analyzed for alanine transaminase (ALT), aspartate transaminase (AST), total protein (TP), albumin (ALB), alkaline phosphatase (ALP), total cholesterol (TCHO), and high-density lipoprotein cholesterol (HDLC) using an autoanalyzer (Dri-chem 4000i®, Fuji, Japan).

### 2.4. Histopathology and Immunohistochemistry (IHC)

Liver tissues were obtained from the left lateral and right medial lobes of each mouse. Tissues obtained were immediately fixed in 10% neutral-buffered formalin and processed routinely with a graded ethanol series for dehydration and with xylene for clearing. The tissues were then embedded in paraffin wax, and 4-*μ*m sections of the paraffinized tissues were stained with hematoxylin and eosin (H&E). To examine the degree of inflammation, all inflammatory lesions around the central veins (CVs) were graded and scored at a microscopic magnification of 200× based on the criteria in Table S2. The scores were totaled for each mouse, and the representative score of each group was presented as the mean ± standard deviation (S.D.). Liver proliferation was estimated by the number of mitotic figures, which were counted in 20 high-power fields (HPFs; 400×); these values are presented as mean ± S.D. for each group. For IHC analysis, endogenous peroxidase activity of the tissue was inhibited using 3% hydrogen peroxide solution in methanol for 20 min. After using the blocking serum, anti-CD68, anti-PCNA, and anti-SMP30 antibodies (Santa Cruz, CA, USA) were applied. Antigen-antibody interactions were visualized with an avidin-biotin peroxidase complex solution from an ABC kit (Vector Laboratories, CA, USA) and DAB substrate (Invitrogen, MA, USA). The tissues were counterstained with hematoxylin.

### 2.5. Western Blot Analysis

Liver tissues were frozen immediately at −80°C until use. For protein analysis, the liver was ground in RIPA buffer containing a protease inhibitor cocktail (Thermo Scientific, MA, USA). The lysate was centrifuged to remove any solid tissue and debris, and the Bradford method employed to quantify the level of protein. The protein samples were loaded and separated by SDS-polyacrylamide gel electrophoresis. After transfer to a polyvinylidene difluoride membrane, 3% bovine serum albumin solution was used to block nonspecific reactions, and anti-CYP2E1, anti-PCNA, anti-SMP30 (Santa Cruz, CA, USA), and anti-GAPDH antibodies (Cell Signaling, MA, USA) used for protein detection. The reaction was developed with an enhanced chemiluminescence reagent (GE Healthcare, UK); thereafter, positive reaction was quantified using Image lab software (Bio-rad, CA, USA).

### 2.6. Statistical Analysis

Results are expressed as mean ± S.D., and data were analyzed using the Student's* t*-test. Statistical significance was assumed when *p* was either < 0.05 or < 0.01.

## 3. Results

### 3.1. Effect of Nutria Bile Administration on Body Weight and Serum Chemistry of Mice with TAA-Induced Liver Injury

**Figure 1:**
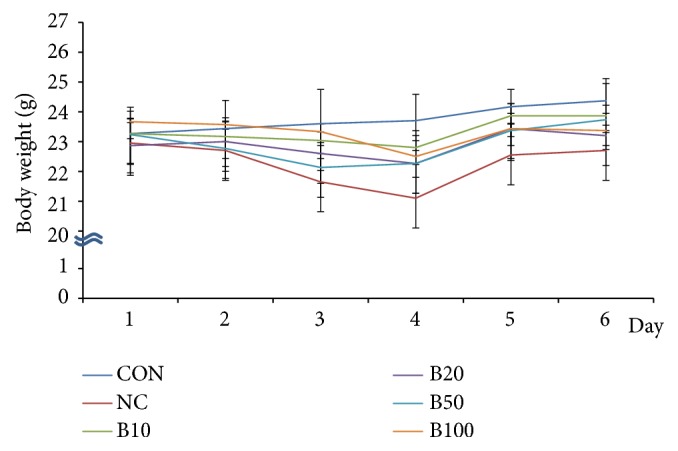
*Effect of bile administration on body weight*. Values are expressed as mean ± S.D.

We measured the body weights of mice on a daily basis and found a decrease following TAA injection in all groups except the CON group. This decrease continued up to day 3 or 4, but from day 5, an increase was observed. On day 6, a body weight change of +1.1 ± 0.2 g in the CON group and -0.3 ± 0.3 g in the NC group was evident compared to baseline (day 1). In addition, on day 6, groups B10, B20, and B50 gained 0.6 ± 0.2 g, 0.3 ± 0.1 g, and 0.5 ± 0.7 g, respectively, compared to day 1. In contrast, a body weight change of -0.3 ± 0.7 g was observed in the B100 group (Figure 1).

**Figure 2:**
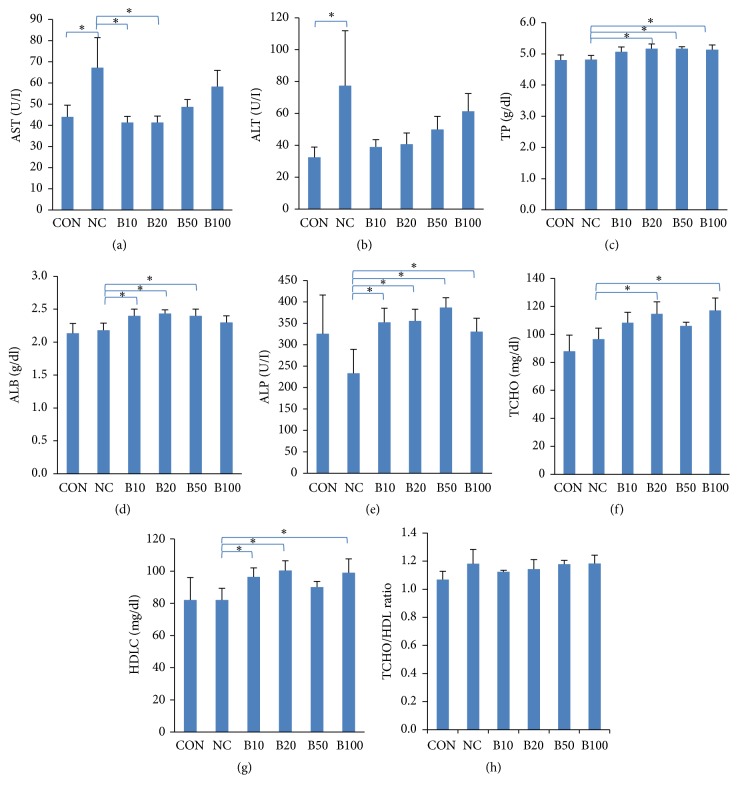
*Effect of bile administration on serum parameters in TAA-induced liver injury.* (a, b) Serum AST and ALT levels. (c-e) Serum TP, ALB, and ALP levels. (f-h) Serum TCHO, HDLC levels and TCHO/HDLC ratio. Values are expressed as mean ± S.D; *∗p* < 0.05 compared to NC.

Mean activities of ALT and AST (indicators of liver damage) were significantly elevated in NC, but lowered in the bile treatment groups. In addition, a significant decrease was observed in the concentration of AST in the B10 and B20 groups (Figures 2(a) and 2(b)). Serum TP and ALB (characteristics of liver function) were unchanged in NC compared to CON; however, in the bile treatment groups, mean TP and ALB levels increased compared to those in NC. Moreover, a significant increase in the serum TP level was found in the B20, B50, and B100 groups, while serum ALB significantly increased in the B10, B20, and B50 groups (Figures 2(c) and 2(d)). Serum ALP activity decreased in NC but recovered significantly (near the normal value) in all treatment groups (Figure 2(e)). Lipid metabolic changes were estimated by measuring the concentration of TCHO and HDLC. As the synthesis of bile acid is the major pathway underlying cholesterol catabolism in mammals, with bile acid as the end product, we expected that total TCHO would increase in the bile treatment groups since there was a decrease in cholesterol utilization to synthesize bile acid. As expected, TCHO increased in the bile treatment groups; however, HDLC was also significantly increased compared to that in NC (Figures 2(f) and 2(g)). Consequently, the TCHO/HDLC ratio, an index of cardiovascular risk, showed no significant difference between all groups (Figure 2(h)). Although there was no significant change in the TCHO/HDLC ratio, HDLC concentrations showed the tendency to increase in response to bile treatment; such increase was significant in B10, B20, and B100 relative to NC. HDLC exerts antiatherogenic effects by reversing cholesterol transport from the peripheral tissues to the liver. In addition, increased HDLC correlates with a lowered risk of cardiovascular diseases [22].

### 3.2. Effect of Nutria Bile Administration on Histopathologic Changes in TAA-Induced Liver Injury

**Figure 3:**
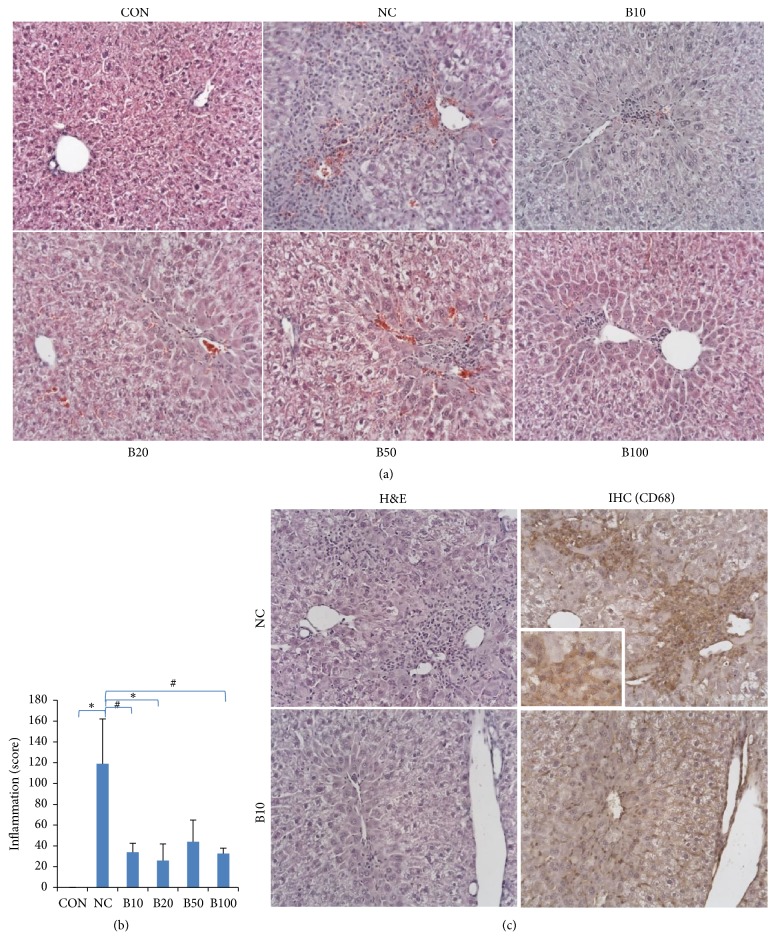
*Effect of nutria bile administration on the histopathology in TAA-induced liver injury.* (a) H&E staining of liver tissues. Centrilobular necrosis and mononuclear cell infiltration were increased by TAA injection, but decreased by bile administration. Original magnification: 200×. (b) Inflammation score. Bile treatment groups had a significant decrease in inflammation compared to NC. (c) H&E staining and IHC (CD68) of liver tissues. Mononuclear cells in inflammation areas of NC were positive to CD68. The number of positive cells dramatically decreased in B10. Original magnifications: 200×, 400× (inset). Values are expressed as mean ± S.D. *∗p* < 0.05 and #*p* < 0.01 compared to NC.

Liver tissues were examined to evaluate histopathologic changes caused by injecting TAA and administering bile (Figure 3(a)). Centrilobular necrosis and mononuclear cell infiltration were induced by TAA. TAA injection led to various degrees of inflammation and necrotic changes in all groups, except CON. In addition, infiltration of mononuclear cells with oval-shaped basophilic nuclei was observed around the CVs. We proceeded to evaluate the inflammation score of each group, as shown in Table S2. Inflammation was most significant in NC (score= 119) and decreases to 26–44 following nutria bile administration found in the treatment groups; this decrease was significant in B10, B20, and B100 compared to NC (Figure 3(b)). To identify mononuclear cells around CVs, IHC was performed. Kupffer cell marker (CD68) was visualized in the cytoplasm of mononuclear inflammatory cells as a deep brown color in NC, but in all bile treatment groups, the number of positive cells was reduced (Figure 3(c)). To add, the mononuclear cells showed a negative reaction to the lymphocyte markers, CD3 and CD79a (data not shown).

### 3.3. Effect of Nutria Bile Administration on CYP2E1 Expression in TAA-Induced Liver Injury

**Figure 4:**
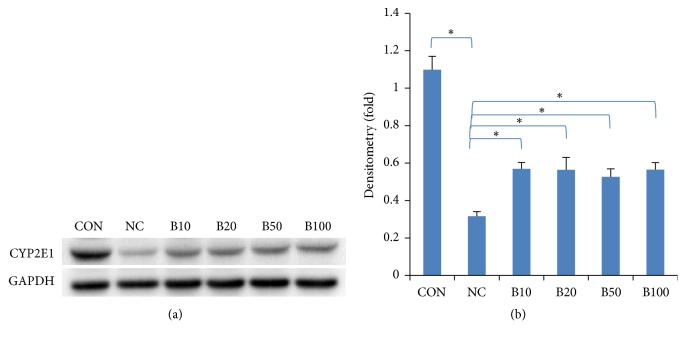
*Effect of nutria bile administration on CYP2E1 expression in TAA-induced liver injury.* (a, b) Western blot analysis and quantification of CYP2E1 expression. CYP2E1 expression level decreased by TAA injection, but, in the bile treatment groups, a significant increase was found compared to NC. Values are expressed as mean ± S.D; *∗p* < 0.05 compared to NC.

CYP2E1 mediates TAA-induced liver injury. To determine whether there was an enzymatic change in TAA, the expression level of CYP2E1 was analyzed by western blot analysis. Its expression dramatically decreased in NC and significantly increased in all bile treatment groups (Figure 4).

### 3.4. Effect of Nutria Bile Administration on Hepatocyte Proliferation in TAA-Induced Liver Injury

**Figure 5:**
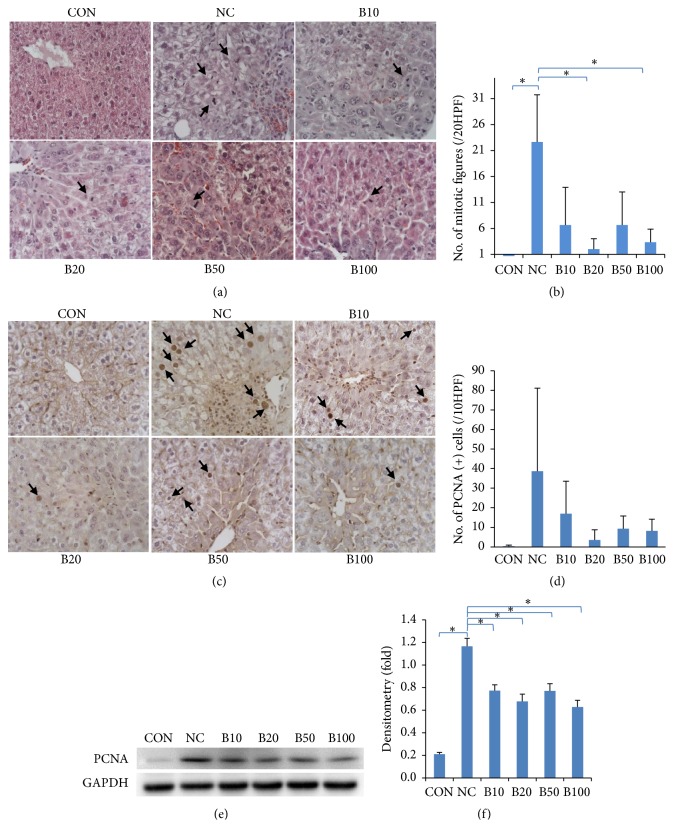
*Effect of nutria bile administration on hepatocyte proliferation in TAA-induced injury.* (a) H&E staining of liver tissues. The number of mitotic figures (arrows) was elevated following TAA injection but was reduced in response to bile administration. Original magnification: 400×. (b) Comparison of the number of mitotic figures calculated in 10 HPF via microscopy. Bile treatment groups had a significant decrease in the number of mitotic figures compared to NC. (c) IHC of PCNA in liver tissues. PCNA-positive hepatocytes (arrows) were elevated following TAA injection but declined in response to bile treatment. Original magnification: 400×. (d) Comparison of the number of PCNA-positive cells counted in 10 HPF by microscopy. Bile treatment groups showed a decrease in proliferative response compared to NC. (e, f) Western blot analysis and quantification of PCNA expression. All bile treatment groups showed a significant decrease in PCNA expression compared to NC. Values are expressed as mean ± S.D. *∗p* < 0.05 compared to NC.

To evaluate hepatocyte proliferation following bile administration, we evaluated the mitotic activity in hepatocytes. Mitotic figures were mainly observed in mid-zonal areas, and, compared to NC, the number of these figures decreased in the bile treatment groups (Figure 5(a)). Mean number of mitotic figures was 22.7 in NC; this decreased to less than 7 in all bile treatment groups, with a statistical significance confirmed in B20 and B100 compared to NC (Figure 5(b)).

The nuclear protein, PCNA, whose levels increase during cell proliferation, was visualized using IHC and quantified by western blot analysis. IHC revealed PCNA-positive hepatocytes around the CVs and mid-zonal areas (Figure 5(c)). Mean positive cells mainly appeared in NC (38.7/10 HPF). The number of PCNA-positive cells was lower in the bile treatment groups than in NC. Although the number of positive cells was 17 in B10, a decrease to 3.50, 9.33, and 8.17 per 10 HPF was found in B20, B50, and B100, respectively (Figure 5(d)). Western blot analysis revealed the dramatic increase in PCNA expression in NC compared to that in CON. Bile administration significantly lowered this expression in all treatment groups (Figures 5(e) and 5(f)).

### 3.5. Effect of Nutria Bile Administration on SMP30 Expression in TAA-Induced Liver Injury

**Figure 6:**
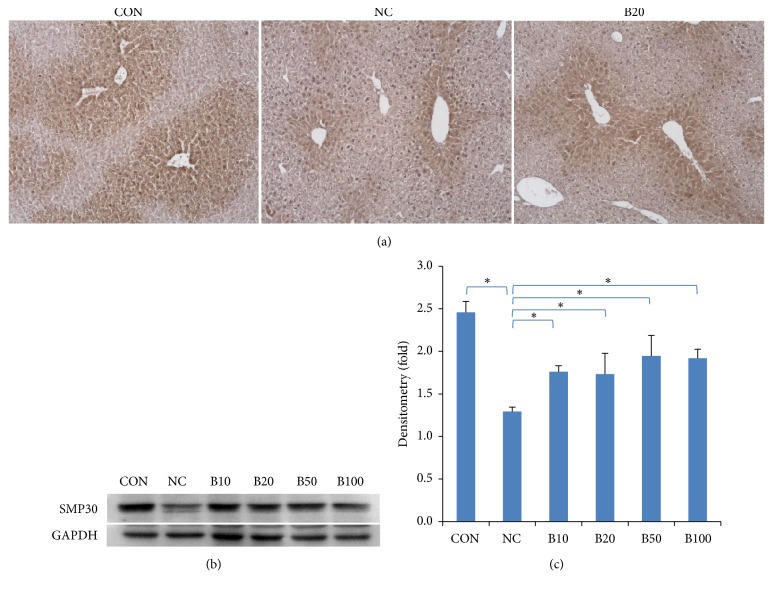
*Effect of nutria bile administration on SMP30 expression in TAA-induced liver injury.* (a) IHC results of SMP30 in liver tissues. SMP30-positive hepatocytes around the CVs decreased following TAA injection, but recovered due to bile administration. Original magnification: 100×. (b, c) Western blot analysis and quantification of SMP30 expression. All bile treatment groups had a significant increase in SMP30 expression compared to NC. Values are expressed as mean ± S.D.; *∗p* < 0.05 compared to NC.

To determine the viability of senescence marker protein 30 (SMP30), which decreases in injured hepatocytes, we performed IHC and western blot analysis. IHC showed that SMP30 localized to the cytosol and nucleus of hepatocytes around CVs. In addition, immunoreactivity was dramatically decreased in NC compared to CON, but SMP30 increased in the treatment groups compared to NC (Figure 6(a)). Quantitative analysis using western blot also confirmed a half-fold reduction in SMP30 expression in response to TAA injection in NC compared to CON. The expression was significantly increased in all bile treatment groups compared to NC (Figures 6(b) and 6(c)).

## 4. Discussion

In the present study, we confirmed the hepatoprotective effect of nutria bile through several experiments. The loss in body weight caused by TAA-induced liver injury recovered more rapidly in the bile treatment groups than in NC. In addition, serum chemistry analysis of changes in ALT, AST, TP, ALB, ALP, and HDLC showed that bile ameliorated liver injury and recovered liver function against TAA-induced hepatotoxicity. We also found a decrease in TAA-induced infiltration of inflammatory cells, mainly Kupffer cells, following the administration of nutria bile.

To identify the underlying mechanism in the regulation of TAA metabolism by bile treatment, western blot analysis was conducted to quantify CYP2E1. Reports have proposed that CYP2E1 is inactivated by TAA or carbon tetrachloride (CCl_4_) and recovered by treatment with hepatoprotective agents [23, 24]. In the present study, CYP2E1 expression decreased in NC and recovered significantly in the bile treatment groups. To estimate hepatocyte proliferation following TAA-induced injury, we examined the number of mitotic figures and PCNA expression in hepatocytes. The number of mitotic figures, which increases in the M phase of mitosis, is a histopathological marker of cell proliferation [25]. PCNA is an accessory protein of DNA polymerase and its level increases in the S phase of mitosis relative to DNA replication [26]. Interestingly, hepatocyte proliferation has been shown to increase in response to TAA-induced injury as a compensatory mechanism to repair damaged liver tissue and decrease in response to treatment with hepatoprotective reagents [27, 28]. In the present study, the number of mitotic figures and PCNA expression was significantly decreased in the bile treatment groups compared to NC. To elucidate the mechanisms by which bile exerts hepatoprotective effects within the bile treatment groups (despite a decrease in hepatocyte proliferation), we examined the expression of SMP30. The expression level of SMP30 is reduced in the liver of older mice (24-month-old) when compared to young mice (3-month-old) [29]. It is also reduced in response to treatment with hepatotoxic agents such as CCl_4_, reactive oxygen species, and 3, 3′, 5-triiodo-L-thyronine (T_3_) [30–32]. SMP30 protects hepatocytes from tumor necrosis factor-*α*- and fas-mediated apoptosis [33]. In the present study, SMP30 concentrations decreased in the NC group but significantly increased in the bile treatment groups. These results demonstrate the reduction in injury caused by TAA in the bile treatment groups.

We confirmed the hepatoprotective effect of nutria bile in a TAA-induced injury model of mice. Serum ALT and AST levels were increased in the groups administered a high dose of bile (B50, B100); these levels, however, were still lower than those in NC. This finding suggests that a high concentration of nutria bile may cause liver damage. Bile acids have been reported to induce cell injury through inflammation, apoptosis, and oxidative stress at high doses or in cholestasis [34, 35]. Song et al. [34] showed that administering UDCA to mice did not increase serum ALT, while other types of bile acids (CA, CDCA, DCA, LCA) resulted in dose-dependent hepatotoxicity. Increases in ALT and AST in groups administered a high dose of bile cannot be explained by the presence of CDCA or 7-keto-LCA in nutria bile alone. Watanabe et al. [18] suggested that CA, but not CDCA, in bile increases AST and ALT activities in mouse models of liver injury. Nonetheless, CDCA has been confirmed as the main farnesoid X receptor (FXR) ligand, and FXR agonists are proposed treatments for cholestatic diseases, including PBC, nonalcoholic fatty liver disease, and portal hypertension [36]. Furthermore, 7-keto-LCA is synthesized as a primary bile acid in nutria and is an intermediate in the hepatic transformation of CDCA to UDCA. [11]. In humans and animals that cannot synthesize UDCA in the liver, 7-keto-LCA is a secondary bile acid found in feces and is formed from CDCA by intestinal bacterial enzymes [37]. The ingestion of 7-keto-LCA by humans has been shown to decrease the biliary lithogenic index, indicating its effectiveness for dissolving gallstones [37]. Bile acid synthetic pathways and their regulation are extremely complex and require further understanding. Nonetheless, administering exogenous bile acid may suppress endogenous bile acid production and biliary cholesterol secretion, and such suppression may cause side effects. A high concentration of nutria bile may therefore cause liver injury, indicating the need for further research to determine appropriate therapeutic concentrations to prevent such injury.

## 5. Conclusion

Although several eradication programs have been implemented worldwide to prevent the introduction and establishment of invasive species, only few studies have focused on the usability of their remains after eradication. We obtained UDCA-rich bile from nutria that was euthanized in an eradication program. This bile exhibited hepatoprotective effects against TAA-induced liver injury in mice. We conclude that although a further study is required to elucidate the molecular mechanisms underlying the therapeutic effects of nutria, bile from their carcasses can be used as a hepatoprotective agent.

## Figures and Tables

**Table 1:** Bile acids composition of a mixture of bile from four wild nutrias.

Bile acids	Content	
	Mean (mg/g)*∗*	Ratio (%)*∗*
Ursodeoxycholic acid (UDCA)	339.7 ± 66.8	34.8 ± 6.8
Chenodeoxycholic acid (CDCA)	431.0 ± 19.7	44.2 ± 2.0
7-ketolithocholic acid (7-keto-LCA)	205.2 ± 40.8	21.0 ± 4.2
Hyodeoxycholic acid (HDCA)	- (trace)	-
7-ketodeoxycholic acid (7-keto-DCA)	- (trace)	-
Deoxycholic acid (DCA)	- (trace)	-
Lithocholic acid (LA)	- (trace)	-

Total	975.9	100.0

*∗* Mean ± SD.

## Data Availability

All data used to support the findings of this study are included within the article and are available from the corresponding author upon request.

## Supplementary Materials

Supplementary Materials Table S1: experimental design of thioacetamide-induced mouse liver injury model. Table S2: grade and score of inflammation in thioacetamide-induced liver injury.
