# Supplementary material for: Protective Effects of Nutria Bile against Thioacetamide-Induced Liver Injury in Mice
Source: Evid Based Complement Alternat Med. 2019 Jun 25;2019:6059317. doi: 10.1155/2019/6059317 (PMC6614960; doi:10.1155/2019/6059317)
Supplement: Supplementary Materials — Table S1: experimental design of thioacetamide-induced mouse liver injury model. Table S2: grade and score of inflammation in thioacetamide-induced liver injury. [file 6059317.f1.pdf]

Supplementary Materials

Table S1. Experimental design of thioacetamide-induced mouse liver injury model.

Group		Thioacetamide (TAA) (IP, mg/kg)	Bile (PO, mg/kg)
Normal control group	(CON)	–	– (0.9% NS)
Negative control group	(NC)	50	– (0.9% NS)
Bile treatment groups	(B10)	50	10
	(B20)	50	20
	(B50)	50	50
	(B100)	50	100

IP, intraperitoneal; PO, per oral; NS, normal saline

Table S2. Grade and score of inflammation in thioacetamide-induced liver injury

Grade	Score	Description of the lesion
Minimal	1	A small cluster of inflammatory cells immediately around CV
Mild	2	A large cluster of inflammatory cells around CV and spreading into midzonal area
Moderate	3	A bridging cluster of inflammatory cells bridging affected CVs
Severe	4	A broad infiltration of inflammatory cells obscuring recognition of hepatic lobular structure

CV, central vein
